# Molecular Characterization of Sudanese and Southern Sudanese Chicken Breeds Using mtDNA D-Loop

**DOI:** 10.1155/2014/928420

**Published:** 2014-04-24

**Authors:** Charles E. Wani, Ibrahim A. Yousif, Muntasir E. Ibrahim, Hassan H. Musa

**Affiliations:** ^1^Department of Genetics and Animal Breeding, Faculty of Animal Production, University of Khartoum, Sudan; ^2^Faculty of Veterinary Sciences, University of Bahr El Ghazal, Sudan; ^3^Institute of Endemic Diseases, University of Khartoum, Sudan; ^4^Faculty of Medical Laboratory Sciences, University of Khartoum, Sudan

## Abstract

The objective of this study was to assess the genetic relationships and diversity and to estimate the amount of gene flow among the five chicken populations from Sudan and South Sudan and commercial strain of egg line White Leghorn chickens. The chicken populations were genotyped using mtDNA D-loop as a molecular marker. PCR product of the mtDNA D-loop segment was 600 bp and 14 haplotypes were identified. The neighbor-joining phylogenetic tree indicated that the indigenous Sudanese chickens can be grouped into two clades, IV and IIIa only. Median joining networks analysis showed that haplotype LBB49 has the highest frequency. The hierarchal analysis of molecular variance (AMOVA) showed that genetic variation within the population was 88.6% and the differentiation among the population was 11.4%. When the populations was redefined into two geographical zones, rich and poor Savanna, the results were fractioned into three genetic variations: between individuals within population 95.5%, between populations within the group 0.75%, and genetic variation between groups 3.75%. The pair wise *F*_st_ showed high genetic difference between Betwil populations and the rest with *F*_st_ ranging from 0.1492 to 0.2447. We found that there is large number of gene exchanges within the Sudanese indigenous chicken (Nm = 4.622).

## 1. Introduction

Chicken genetic resources comprise a wide range of breeds and populations including red jungle fowl, native and fancy breeds, middle level food producers, industrial stocks, and specialized lines. Sudan possesses potential genetic resources of local poultry, even though most of them have not been genetically and phenotypically characterized. Some efforts had been exerted by the Sudanese nationals [1, 2] and international researchers and FAO investigated the genetic and phenotypic potentiality of the local chickens but still further identification and characterization as an ultimate prerequisite for their conservation and utilization are needed. The assessment of genetic distance by means of molecular marker techniques may provide useful information for initial evaluation of chicken genetic resources. Microsatellites have been successfully used in chicken genetic diversity studies. Genetic diversity measures using the highly polymorphic variable number of tandem repeat loci have yielded reliable and accurate information for the study of genetic relationships between chicken populations. Sequencing a specific fragment of mtDNA (e.g., D-loop) gives more accurate information on evolution and genetic diversity [3]. The D-loop region does not encode protein and evolves much faster than other region of the mtDNA genome. For the past 20 years, mtDNA and particularly D-loop sequences have been used in phylogenetic analysis [4]. There is evidence from mtDNA D-loop variations in European, African, and Indian cattle breeds that indicate independent domestications of* Bos taurus *and* Bos indicus *cattle in two separate locations [5]. Also D-loop sequences have been used in unraveling domestication and diversity of dogs [6], horses [7], and goats [8] and in Australian red kangaroo and* Macropus rufus *[9]. Study variation among 398 African indigenous chicken from 12 countries was carried out using mtDNA D-loop region and HVI domain and it was found that the polymorphic sites account for 12.59% of the 397 sequenced base pair fragment [10], while variation rates among 25 individuals from six native Chinese chicken populations were recorded to be 7.05% and 5.54%, respectively [11, 12]. Advantages of the mtDNA are that it plays a role in metabolism, apoptosis, disease, and aging and it is the site of oxidative phosphorylation essential for the production of ATP, and a variety of other biochemical functions. It is highly polymorphic compared to the nuclear DNA evolutionary rate being 5–10 times faster than nuclear genome [13] probably due to lack of replication repair mechanism [14]. The understanding of phylogeography will elucidate the demographic history, origin, and expansion of livestock species. Networks analysis has supplemented phylogenetic trees to overcome the problem of parallel mutations and lineage exchange between divergent populations [15]. Therefore, the aim of the present study is to evaluate the genetic variability within and between Sudanese native chickens using mtDNA D-loop region.

## 2. Materials and Methods

### 2.1. Experimental Animals

The study was conducted in four states of Sudan, chosen because they have only indigenous chicken and no exotic breeds have been introduced. A total of 81 blood samples were collected from five chicken populations including Betwil, (BTW, *n* = 21) from El Dilling Locality in South Kordofan State, and Large Beladi of Bhari (LBB, *n* = 12) was collected from Khartoum North Locality. Large Beladi of Abu-Neama (LB, *n* = 22) and Bare Neck (BRN, *n* = 12) were both collected from Abu-Neama Locality in Sinnar State. The fifth population was (SUD, *n* = 14) from Malakal Locality; this sample was retrieved from International livestock Research Institute (ILRI) data base. In addition to a commercial strain of egg line White Leghorn type (COML *n* = 9) was genotyped concurrently with the Sudanese chickens.

*Genetic Analysis*. DNA was extracted using the Chloroform Method [16]. PCR was performed in 30 *μ*L reaction containing 2.5 mM of each dNTPs, 14 pmol of each primer, 1.5 mM MgCl_2_, 1 × PCR buffer, 1.25U Taq DNA polymerase (Roche Applied Sciences, Germany or Promega, Madison, USA), and 1 *μ*L Genomic DNA. The mtDNA D-loop (HV1) region was amplified using specific primers based on the partial chicken mitochondrial genome GenBank accession number (AB098668) and complete chicken mitochondrial genome GenBank accession number (NC 001323) (Table 1). PCR amplification was carried out on a Gene Amp PCR 9700 (Applied Biosystems) thermo cycler. PCR conditions were as follows: initial denaturation at 94°C for 2 min, followed by 10 cycles at 94°C for 15 s, 58°C for 30 s, and 72°C for 40 s. The amplified fragments were electrophoreses on 1.5% agarose gel at 100 volts for one hour; gel was stained with 0.4 *μ*L/mL ethidium bromide and detected under UV light. PCR products were purified using QIAquick PCR purification kit (QIAGEN GmbH, Germany). Direct sequence of HV1 segment of D-loop region was performed using two internal primers CR-for and CR-rev as shown in Table 1. The sequence was done using the BioDye Terminator version 3.1 cycle sequencer kit (Applied Biosystems) with total volume of 25 *μ*L comprising 20 ng of purified PCR product as template DNA and 3.2 pmol of primer.

### 2.2. The mtDNA Data Analysis

The mtDNA sequence for the first nucleotides (600 bp) of D-loop region [17] was used for analysis after editing the sequences of amplified D-loop fragments. Multiple alignments of the sequences were performed using ClustalX 1.83 [18] and Muscle 3.52 [19] programs.

### 2.3. Phylogenetic and Molecular Evolution Analysis

The evolutionary relationships of indigenous Sudanese chicken haplotypes were established using the molecular evolutionary genetics analysis (MEGA) version 3.0 [20]. Genetic distances of the haplotypes were calculated using Kimura's two parameters model to construct a neighbor joining phylogenetic tree. Two D-loop sequences of* Gallus* were included, one from wild jungle fowl* Gallus gallus gallus *and the other from* Gallus gallus bankiva *GenBank accession (number AB007720 and AB007718), respectively, and seven Clade I, II, IIIa, IIIb, IIIc, IIId, and IV reference sequences that correspond to different clades determined previously in Asian samples [10] (Table 2).

### 2.4. Haplotypes Diversity

Haplotype diversity was illustrated using network analysis implemented by NETWORK 4.1.0.8 [15]. The DNA D-loop sequences diversity indices were determined to elucidate the sequence polymorphism and the content of genetic variability in chicken population. The populations indices include number of segregation sites (*S*), number of haplotypes (*H*), haplotype diversity (Hd), and nucleotide diversity (*π*) as explained by Nei [21]. The analysis was conducted using DnaSP software version 4.0 [22]. Alignment gaps arising from a deletion event were excluded from the calculations. The average number of nucleotides differences per site between the two sequences known as nucleotide diversity (*π*) is defined as *π* = *n*/(*n* − 1)Σ*x*_*i*_ *x*_*j*_ *π*_*ij*_ or *π* = Σ*π*_*ij*_/*n*_*c*_ where *n* is the number of DNA sequences examined, *x*_*i*_ and *x*_*j*_ are the frequencies of the *i*th and *j*th type of DNA sequences, respectively, in the sample, *π*_*ij*_ is the proportion of nucleotides in the respective types of DNA sequences, and *n*_*c*_ is the total number of sequence comparisons [21]. Average heterozygosity or haplotype diversity, *h*, is defined according to the formula of Nei [21], *h* = 2*n*(1 − Σ*x*_*i*_^2^)/(2*n* − 1), where *x*_*i*_ is the frequency of haplotype and *n* is the sample size. The degree of genetic differentiation among the population was estimated using gene or haplotype frequencies. Population genetic structure was investigated by *F*_st_ significance test [23] and *N*_st_ [24] using Arlequin software version 2.000 [25].

### 2.5. Analysis of Molecular Variance (AMOVA)

Maternal genetic differentiation was further quantified using hierarchical analysis of molecular variance, AMOVA [24], performed using Arlequin version 2.000 software [25]. Sudanese chicken population was first considered as one single population and later it was subdivided into two geographical areas, rich and poor Savanna.

## 3. Results

### 3.1. Pattern of mtDNA D-Loop Variability

**Figure 1:**
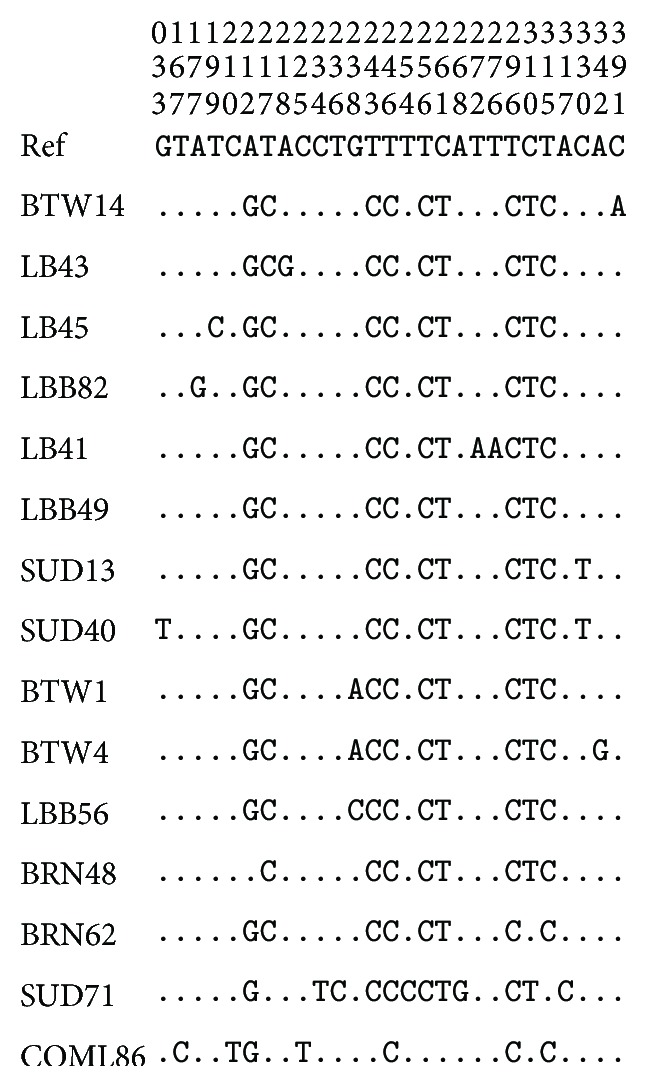
Pattern of mtDNA D-loop variability. Note: nucleotide polymorphisms observed in D-loop HV1 domain of 81 chicken sequences. Vertically oriented numbers indicate the site position and the sequences shown are only the variable sites. Dots (.) indicate identity with the reference sequence and different base letters denote substitution.

The pattern of 600 bp mtDNA D-loop variability revealed high variations between nucleotide 212 and 397. Fourteen haplotypes were identified in the Sudanese and Southern Sudan indigenous chicken populations with variation at 19 sites, and 23.5% of them are polymorphic (Figure 1).

Multiple sequence alignment was performed for the fourteen haplotypes identified in the Sudanese and Southern Sudan indigenous chicken. Alignment of D-loop sequences was done to a reference sequence from Gene Bank accession number (AB 098668) using Clustal-X 1.83. Two units of invariant tetradecamer 5′-AACTATGAATGGTT-3′ were detected at positions 267 to 280 and 328 to 341. In the first unit there were two transitions observed G/A for SUD 71 and T/A for LB41 at position 268 and 272, respectively, while in the second unit of the tetradecamer T/C transition was observed for SUD 40 and SUD 13 at position 330. In addition to that the following domains and motif were observed, at the 5′ end of the D-loop. An interrupted thymine string (AATTTTATTTTTT) was observed and found to be conserved in all the individuals studied. There was also an interrupted poly-C sequence (5′-CCCCCCCTTTCCCCCCC-3′) which is widely conserved and downstream to this there is conserved sequence known as poly-G (5′-AGGGGGGGT-3′). Two conserved 5′-TACAT-3′ and 5′-TATAT-3′ were also found in all individuals. There are six TATAT motifs and two TACAT found within the 397 bases of the D-loop and were also conserved. The first 166 base pairs adjacent to tRNA Glu were found to be highly conserved in all individuals except for one substitution a T/G transversions in SUD 40 at position 33 (data not shown).

The nucleotide substitutions found in the 14 variable haplotypes comprised one G/T and two C/A transversions and the rest were all transitions of which six were A/G substitutions and ten were C/T substitutions. This demonstrates a strong bias towards transition. The C/T substitutions are more common than A/G substitution (Table 3).

### 3.2. Phylogenetic Analysis of the Haplotypes

**Figure 2:**
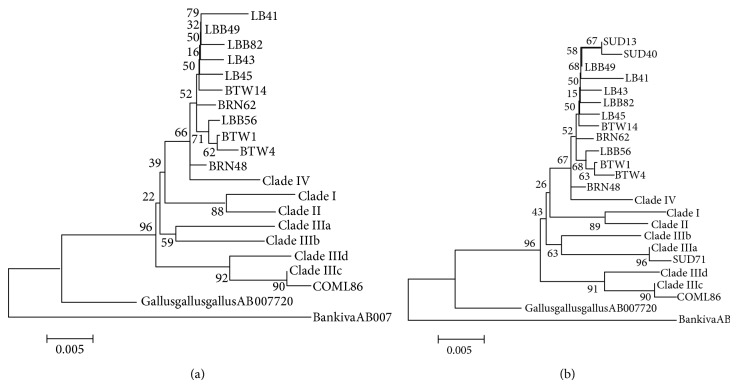
(a) Neighbour-joining tree reconstructed using MEGA 3.1 software from 11 haplotypes identified in Sudanese indigenous chickens, two haplotypes of the genus* Gallus *retrieved from GenBank:* Gallus gallus gallus *(GenBank accession number AB007720) and* Gallus gallus bankiva *(GenBank accession number AB007718), and seven clade reference haplotypes (Clade I, II, IIIa, IIIb, IIIc, IIId, and IV). The numbers at the nodes represent the percentage bootstrap values for interior branches after 1000 replications. (b) Neighbour-joining tree reconstructed using MEGA 3.1 software from 14 haplotypes identified in Sudanese indigenous chickens, two haplotypes of the genus* Gallus *retrieved from GenBank:* Gallus gallus gallus *(GenBank accession number AB007720) and* Gallus gallus bankiva *(GenBank accession number AB007718), and seven clade reference haplotypes (Clade I, II, IIIa, IIIb, IIIc, IIId, and IV). The numbers at the nodes represent the percentage bootstrap values for interior branches after 1000 replications.

A neighbor joining dendrogram showed the genetic relationships among the twelve haplotypes identified in Sudanese indigenous chicken from Sudan. The egg line commercial strain chicken was included and two haplotypes of genus* Gallus* (*Gallus gallus gallus *and* Gallus gallus bankiva*, GenBank accession number AB007720 and AB007718, resp.) were retrieved from GenBank and used as out groups, and seven clade reference haplotypes (Clade I, II, IIIa, IIIb, IIIc, IIId, and IV) were also included. The dendrogram revealed that 11 haplotypes identified in the Sudanese indigenous chicken were placed into two clusters with the domestic chicken* Gallus gallus gallus. *This indicates a very close relationship between the Sudanese indigenous chickens and* Gallus gallus gallus*, while they are relatively genetically distanced from* Gallus gallus bankiva*. Alignment with the reference lineage haplotypes from Asia showedthat all Sudanese indigenous chicken were grouped into clade IV (Figure 2(a)), while the commercial egg line strainstudied concurrently with Sudanese indigenous chicken fell into clade IIIc. When 3 haplotypes from Upper Nile State of the Southern Sudan werecombined with 11 haplotypes from the Sudan and aligned together again with* Gallus gallus gallus *and* Gallus gallus bankiva*, the dendrogram constructed placed Sudanese chicken haplotypes into three clusters with thedomestic haplotype from Genbank. This once more suggest that the Sudanese indigenous chicken is more closely related to* Gallus gallus gallus *while they are relatively genetically distanced from* Gallus gallus bankiva*. Alignment with reference haplotypes from Asia resulted in constructed neighbor-joining tree which grouped the Sudanese indigenous chicken into two, clade IV and clade IIIa, Figure 2(b).

### 3.3. Network Analysis

**Figure 3:**
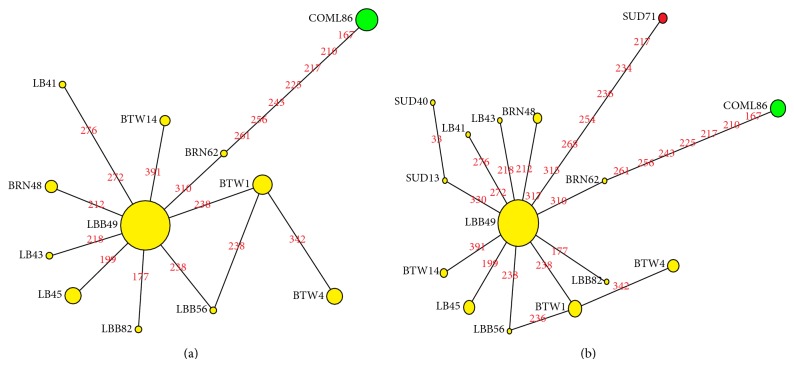
(a) Median-joining network (*ε* = 0) for 11 haplotypes of the northern Sudan indigenous chickens and one haplotype of commercial layers. The yellow circles refer to clade IV while the green circle refers to clade IIIc and the green circle denotes clade IIIc. The size of the circles is proportional to the frequency of the respective haplotypes and the numbers between the haplotype nodes refer to the positions of the nucleotide mutations compared to reference sequence (GenBank accession AB098668). (b) The yellow circles refer to clade IV and the green circle refers to clade IIIc while the red circle denotes clade IIIa.

Median-joining networks were drawn for the 12 haplotypes identified from the Sudanese indigenous chickens from Northern Sudan and one haplotype of commercial layers, based on the variable characters of the complete alignment using the computer program NETWORK 4.1.0.8 [15]. The results showed that DNA sequence of haplotype LBB49 has the highest frequencies and this haplotype is connected to the frequencies of other haplotypes forming star-like connections with LBB49 in the centre. It was also observed that there are mutational links to ten haplotypes which include five singletons. Therefore, it can be referred to as an interior or ancestry haplotype. No median vector (mv)^*^ separating the clade was observed; all the eleven haplotypes identified in the Sudan region belong to clade IV marked with yellow color as shown in Figure 3(a), while the commercial egg line chicken haplotype belongs to different clade which IIIc, marked with green color, and has seven and eight mutation connection with BRN62 and LBB49, respectively.

Median-joining network analysis was carried out with the haplotypes from the Southern States of the Sudan and the Northern Sudan States. The results illustrate that out of the 14 identified Sudanese haplotypes only one haplotype (SUD 71) from the South Sudan (Malakal) showed uniqueness. It fell into a different clade (IIIa) marked with red color while two haplotypes (SUD13 and SUD40) are both sharing clade 1V with other haplotypes from the Sudan region marked with yellow color Figure 3(b).

### 3.4. Population Diversity

The diversity indices were calculated for the five Sudanese indigenous chicken populations from 81 D-loop sequences and 19 segregating sites. The highest number of haplotype (*H* = 6) was found in Large Beladi Abu-Neama populations from Sinnar State, followed by Beladi Malakal chicken from Upper Nile State with (*H* = 5). Betwil, Beladi Bahri, and Bare-Neck had equal number of haplotypes (*H* = 4) and are regarded as the lowest haplotype number. The gene haplotype diversity (Hd) was high in Betwil population (Hd = 0.724) followed by Beladi Malakal while it was lower in Beladi Bahri and Bare Neck population (Hd = 0.455). However, the average overall haplotype diversity was approximately (0.577) for the 81 chicken haplotypes. The average nucleotide diversity detected for 81 D-loop sequences of the indigenous Sudanese chicken population was estimated to be 0.00282 substitutions per site. However, the highest nucleotide diversity was found in Malakal population (0.00603 = *π*) followed by Betwil population (0.00259 = *π*) and Beladi Neama ( = *π*0.00179) while Beladi Bahri and Bare recorded lowest nucleotide diversity (0.00126 = *π*) (Table 4).

### 3.5. Genetic Differentiation

*F*_st_ and *N*_st_ were computed using DnSP version 4.0 [22]; the average *F*_st_ and *N*_st_ were similar (0.098), while Nm was 4.622 for both approaches. This indicates that 9.8% of maternal genetic differentiation estimated in Sudanese indigenous chicken resulted from variation among populations while 90.2% was due to contribution by genetic divergences among individuals within populations. The highest genetic differentiation between populations observed was between Betwil population and the rest of the populations with *N*_st_ value ranging from 0.1493 to 0.2450 while the rest of populations showed a relatively little maternal genetic subdivisions Table 5.

### 3.6. Analysis of Molecular Variance (AMOVA)

Maternal genetic differentiation within population and among population within the Sudanese chicken was quantified using hierarchal analysis of molecular variance AMOVA on Kimura-2-parameter distance considering Sudanese populations as one single group. The genetic variation within population was 88.6% and the genetic differentiation among the populations was 11.4%. When Sudanese chicken population was once more subdivided into two geographical groups: rich and poor Savanna, the resulting variation was partitioned into three fractions. The variation between individuals within populations was 95.5%, between populations within groups was 0.75%, and the genetic variation between groups was 3.75% (Table 6).

## 4. Discussion

The complete alignment revealed a very high variability in the mtDNA D-loop region between 167–391 bases; this variation constitutes 23.5% of the 81 sequences. This rate is extremely high compared to the native chicken breeds 5.54–7.05% [11, 12]. Similarly the results were higher 12.59% than those of 398 African domestic chickens from 12 countries [10]. This high rate mtDNA D-loop variation may be attributed to migration and exploratory movement of human into Sudan being as the largest country in Africa sharing borders with nine countries. The base composition of the Sudanese domestic chicken D-loop HVI shows that A + T sequence content constitutes 50.46% while G + C was 49.54%; similar results were noted by Ruokonen and Kvist [26]. The two units of invariant tetradecamer 5′-AACTATGAATGGTT-3′ which was observed in this study at positions 267 to 280 and 328 to 341 were found to be conserved in most of the 14 haplotypes identified in the Sudanese indigenous chicken except for 4 haplotypes. These four haplotypes were varied by one base substitution. The substitutions were SUD71 with G/A, LB41 with T/C transition at positions 268 and 272, respectively, in the first tetradecamer unit, and SUD13 and SUD40 both with T/C transition at position 330 in the second tetradecamer unit. This type of tetradecamer duplication was also observed by Fumihito et al. [3] and found to be a specific trait for genus* Gallus gallus gallus*. However, this result indicates the close genetic relationship between Sudanese indigenous chicken and genus* Gallus*. On the other hand, at the 5′ end of the D-loop HVI domain an interrupted thymine string (AATTTTATTTTTT), an interrupted poly-C (5′-CCCCCCCTTTCCCCCCC-3′) and poly-G (5′-AGGGGGGGT-3′) were widely conserved in all the individuals. These conserved features have been described across many avian species other than Galliform. They include Struthioniformes, Falconiformes, and Sphenisciformes [27]. However the presence of the cytosines and guanines strings in proximately to each other in D-loop segment sequence of the Sudanese indigenous chicken makes the formation of a stable hairpin structure possible [28]. The conserved sequence motifs of TACAT and TATA were found in all domestic chicken of Sudan. These types of motifs are described as TASs, termination-associated sequences elements involved in the termination of mtDNA synthesis [29]. The presence of the TASs in both Galliformesand mammals may suggest strong structural function of D-loop region of the two genera, while the lack of variation in TASs among the Galliformes may be due to the selective functional constraints. Phylogenetic analysis of the 14 haplotypes identified from the Sudanese indigenous chicken illustrated evolutionary relationships.

All Sudanese chicken population from the Northern states fell into two clusters. However, when a second phylogenetic tree was reconstructed including populations from the Upper Nile State (Malakal) in South Sudan, the dendrogram constructed placed the Sudanese indigenous chicken into three clusters and two different clades meaning that Sudanese chicken came from two different maternal lineages out of the seven clades. Despite the fact that some populations have more than one haplotype yet they fell in the same maternal lineage, that is, all Sudanese different haplotypes fall in Clade IV except haplotype SUD 71 which fell in Clade IIIa, this result may suggest that these populations shared the same maternal ancestor and that their descendants have accumulated mutations to become distinct lineages.

Total nucleotide diversity among the Sudanese chicken was found to be (0.00282 = *π*) nucleotide substitutions per site; it was higher in Malakal and Betwil populations, while it was lower in Beladi Bahri and Bare neck populations. This low nucleotide diversity in Large Beladi of Bhari and Bare neck populations may indicate loss of gene diversity for these populations which may be attributed to relatively recent population bottleneck. On the other hand high nucleotide diversity in Malakal and Betwil populations may suggest that the populations are more ancient [11]. Network analysis showed that DNA sequence of the haplotype of Large Beladi (LBB49) has the highest frequency and connected with the largest number forming a star-like structure. Such pattern of structure was found for different species of birds including Red winged Blackbird, Red poll finches, and Greenfinch [30, 31]. The analysis also revealed convergent or reverse mutation among haplotypes LBB40, LBB56, and BTW1. This convergent mutation is common where there is heterogeneity due to unequal mutation rate for all nucleotide sites. Under such circumstance, accumulation of mutations at a small number of fast sites leads to reverse mutation [32]. Moreover, the network analysis showed that there is probably more than one maternal origin of Sudanese indigenous chicken populations as one haplotype from Malakal population fell into a different (clade IIIa).

The hierarchical analysis of molecular variance AMOVA and *F*_st_ or *N*_st_ significance test indicate that 9.8% of maternal genetic differentiation in Sudanese indigenous chicken populations resulted from variation among populations while 90.2% was due to contribution by genetic divergence among individual within population. The highest observed genetic differentiation between populations was between Betwil population and the rest of the populations followed by Malakal population. The level of *F*_st_ value found in this study is close to the value reported in African cattle breeds (*F*_st_ = 0.060), [33] but smaller than that reported among 78 Chinese indigenous chicken breeds *F*_st_ = 0.106 [34]. When using hierarchical analysis as a second tool to give more insight into genetic differentiation between individuals within the population and to confirm *N*_st_ results, the Sudanese chicken population was first defined into two geographical groups or regions, rich and poor Savanna. The genetic variation between individuals within populations was 95.5% and that occurring between populations within the groups was 0.75%, while the genetic variation between groups accounted for 3.75%. The low genetic variation between the groups that were defined geographically may suggest weak phylogeographic structure of Sudanese chicken and may be an indicator of common maternal origin. When considering Sudanese chicken population as one single population group, the genetic variation within the population accounted for 88.6% of the total variance while the proportion between populations was 11.4%. This indicates that the Sudanese chicken populations are genetically differentiated along geographical localities. Finally the study concludes that the region of mtDNA D-loop HVI which ranges from 167 to 397 has higher variation among Sudanese domestic chicken population.

## Figures and Tables

**Table 1:** Primers used for the amplification and sequencing of HVI segment of the D-loop.

Primer type	Primer name	5′ to 3′ Sequence
PCR primer	L16750 (A)∗	AGGACTACGGCTTGAAAAGC
	H547 (D)	ATGTGCCTGACCGAGGAACCAG
	CR1b (E)	CCATACACGCAAACCGTCTC

Sequencing primer	CR-For (B)	TCTATATTCCACATTTCTC
	CR- Rev (C)	GCGAGCATAACCAAATGG

A, D, E, B, and E indicate annealing point of the primer.

**Table 2:** Reference chicken haplotypes.

Haplotype name	Code of haplotype	Sampling site
Clade I	AF128344∗	China
Clade II	AB009436∗	Lombok Island, Indonesia
Clade IIIa	FL17	Thailand
Clade IIIb	DW07	China
Clade IIIc	DW02	China
Clade IIId	DC15	China
Clade IV	PKD15	Pakistan

**Table 3:** Nucleotide substitutions in D-loop HVI region of indigenous Sudanese and Southern Sudanese chicken.

Substitution	Variable sites
G/T	33
C/A	317 and 391
A/G	177, 212, 218, 238, 268, and 342
C/T	167, 199, 210, 217, 234, 236, 254, 310, 315, and 330

**Table 4:** mtDNA D-loop sequence diversity indices in Sudanese and Southern Sudanese chicken populations based on 397 nucleotides.

Number	Population	*N*	*S*	*H*	Hd	*π*
1	LB	22	6	6	0.47619	0.00179
2	LBB	12	3	4	0.45455	0.00126
3	SUD	14	10	5	0.59341	0.00603
4	BRN	12	3	4	0.45455	0.00126
5	BTW	21	3	4	0.72381	0.00259

	Total	81				

*N*: number of sequences used; *S*: number of segregation sites; *H*: number of hypostyles; Hd: haplotypes diversity; *π*: nucleotide diversity.

**Table 5:** Pair wise *N*_st_ and *F*_st_ values between 5 chicken populations based on D-loop sequences.

	LB	LBB	SUD	BRN	BTW
LB	—	0.0018	0.0514	−0.0108	0.1493
LBB	0.0018	—	0.0536	−0.0285	0.2141
SUD	0.0511	0.0536	—	0.0573	0.1356
BRN	−0.0108	−0.00286	0.0572	—	0.2450
BTW	0.1492	0.2139	0.1351	0.2447	—

Pair wise *N*_st_ values were above the diagonal and Pair wise *F*_st_ values were below the diagonal. LBN: large Beladi Neama; LBB: Large Beladi Bahri; BRN: Bare neck; and BTW: Betwil.

**Table 6:** Analysis of molecular variance (AMOVA) based on partial D-loop sequences of Sudanese and Southern Sudanese indigenous chicken populations.

Samples	Number of groups	Number of populations	Variation (%)		
			Within populations	Among populations within group	Among groups
All 81 chicken populations	2	5	95.5	0.75	3.75
All 5 chicken populations as one group	1	5	88.6	11.41	—
Rich Savanna	1	2	98.3	1.7	—
Poor Savanna	1	3	92.41	7.59	—

Average *F*_st_: 0.09752; Nm: 4.63.

## References

[1] Yousif I. A. (1987). *Phenotypic and genetic variation in body weight of the indigenous chicken [M.S. thesis]*.

[2] Suleiman M. E. F. (1996). *Egg characteristics, genetic and phenotypic relationships of body weight at various ages in indigenous chicken [M.S. thesis]*.

[3] Fumihito A., Miyake T., Sumi S., Takada M., Ohno S., Kondo N. (1994). One subspecies of the red junglefowl (*Gallus gallus gallus*) suffices as the matriarchic ancestor of all domestic breeds. *Proceedings of the National Academy of Sciences of the United States of America*.

[4] Moore W. S. (1995). Inferring phylogenies from mitochondrial DNA variation: mitochondrial-gene trees versus nuclear-gene trees. *Evolution*.

[5] Loftus R. T., MacHugh D. E., Bradley D. G., Sharp P. M., Cunningham P. (1994). Evidence for two independent domestications of cattle. *Proceedings of the National Academy of Sciences of the United States of America*.

[6] Savolainen P., Zhang Y. P., Luo J., Lundeberg J., Leitner T. (2002). Genetic evidence for an East Asian origin of domestic dogs. *Science*.

[7] Jansen T., Foster P., Levine M. A. (2002). Mitochondrial DNA and the origins of the domestic horse. *Proceedings of the National Academy of Sciences of the United States of America*.

[8] Luikart G., Gielly L., Excoffier L., Vigne J.-D., Bouvet J., Taberlet P. (2001). Multiple maternal origins and weak phylogeographic structure in domestic goats. *Proceedings of the National Academy of Sciences of the United States of America*.

[9] Clegg S., Hale P., Moritz C. (1998). Molecular population genetics of the red kangaroo (*Macropus rufus*): mtDNA variation. *Molecular Ecology*.

[10] Mobegi A. V. (2006). *Genetic characterization of African chicken using mitochondrial DNA D-loop sequences [M.S. thesis]*.

[11] Liu Z. G., Lei C. Z., Luo J., Ding C., Chen G. H., Chang H., Wang K. H., Liu X. X., Zhang X. Y., Xiao X. J., Wu S. L. (2004). Genetic variability of mtDNA sequences in chinese native chicken breeds. *Asian-Australasian Journal of Animal Sciences*.

[12] Niu D., Fu Y., Luo J., Ruan H., Yu X.-P., Chen G., Zhang Y.-P. (2002). The origin and genetic diversity of Chinese native chicken breeds. *Biochemical Genetics*.

[13] Brown W. M., Prager E. M., Wang A., Wilson A. C. (1982). Mitochondrial DNA sequences of primates: tempo and mode of evolution. *Journal of Molecular Evolution*.

[14] Clayton D. A. (1982). Replication of animal mitochondrial DNA. *Cell*.

[15] Bandelt H. J., Foster P., Rohl A. (1999). Median-joining networks for inferring intraspecific phylogenies. *Molecular Biology and Evolution*.

[16] Sambrook J., Fritsch E. F., Maniatis T. (1989). *Molecular Cloning: A Laboratory Manual*.

[17] Buehler D. M., Baker A. J. (2003). Characterization of the red knot (*Calidris canutus*) mitochondrial control region. *Genome*.

[18] Thompson J. D., Gibson T. J., Plewniak F., Jeanmougin F., Higgins D. G. (1997). The CLUSTAL_X windows interface: flexible strategies for multiple sequence alignment aided by quality analysis tools. *Nucleic Acids Research*.

[19] Edgar R. C. (2004). MUSCLE: multiple sequence alignment with high accuracy and high throughput. *Nucleic Acids Research*.

[20] Kumar S., Tamura K., Nei M. (2004). MEGA3: integrated software for Molecular Evolutionary Genetics Analysis and sequence alignment. *Briefings in Bioinformatics*.

[21] Nei M. (1987). *Molecular Evolution Genetics*.

[22] Rozas J., Sanchez-DelBarrio J. C., Messeguer X., Rozas R. (2003). DnaSP, DNA polymorphism analyses by the coalescent and other methods. *Bioinformatics*.

[23] Wright S. (1951). The genetical structure of populations. *Annals of Eugenics*.

[24] Excoffier L., Smouse P., Quattro J. (1992). Analysis of molecular variance inferred from metric distances among DNA haplotypes: application to human mitochondrial DNA restriction data. *Genetics*.

[25] Schneider S., Roessli D., Excoffier L. (2000). *Arlequin Version 2.000: Software for Population Genetics and Data Analysis*.

[26] Ruokonen M., Kvist L. (2002). Structure and evolution of the avian mitochondrial control region. *Molecular Phylogenetics and Evolution*.

[27] Haring E., Kruckenhauser L., Gamauf A., Riesing J. M., Pinsker W. (2001). The complete sequence of the mitochondrial genome of *Buteo buteo* (Aves, Accipitridae) indicates an early split in the phylogeny of raptors. *Molecular Biology and Evolution*.

[28] Quinn T. W., Wilson A. C. (1993). Sequence evolution in and around the mitochondrial control region in birds. *Journal of Molecular Evolution*.

[29] Foran D. R., Hixon J. E., Brown W. M. (1988). Comparisons of ape and human sequences that regulate mitochondrial DNA transcription and D-loop DNA synthesis. *Nucleic Acids Research*.

[30] Seutin G., Ratcliffe L. M., Boag P. T. (1995). Mitochondrial DNA homogeneity in the phenotypically diverse redpoll finch complex (Aves: Carduelinae: *Carduelis flammea-hornemanni*). *Evolution*.

[31] Merila J., Bjorklund M., Baker A. J. (1997). Historical demography and present day population structure of the greenfinch, *Carduelis chloris*—an analysis of mtDNA control-region sequences. *Evolution*.

[32] Wakeley J. (1993). Substitution rate variation among sites in hypervariable region 1 of human mitochondrial DNA. *Journal of Molecular Evolution*.

[33] Ibeagha-Awemu E. M., Erhardt G. (2005). Genetic structure and differentiation of 12 African *Bos indicus* and *Bos taurus* cattle breeds, inferred from protein and microsatellite polymorphisms. *Journal of Animal Breeding and Genetics*.

[34] Qu L. J., Li X. Y., Xu G. E. (2006). Evaluation of genetic diversity in Chinese indigenous chicken breeds using microsatellite markers. *Science in China Series C: Life Sciences*.

